# Depression and cardiovascular risk—association among Beck Depression Inventory, PCSK9 levels and insulin resistance

**DOI:** 10.1186/s12933-020-01158-6

**Published:** 2020-11-03

**Authors:** C. Macchi, C. Favero, A. Ceresa, L. Vigna, D. M. Conti, A. C. Pesatori, G. Racagni, A. Corsini, N. Ferri, C. R. Sirtori, M. Buoli, V. Bollati, M. Ruscica

**Affiliations:** 1 Department of Pharmacological and Biomolecular Sciences, Università degli Studi di Milano, Milan, Italy; 2 EPIGET Department of Clinical Sciences and Community Health, Università degli Studi di Milano, Milan, Italy; 3 Department of Pathophysiology and Transplantation, University of Milan, Milan, Italy; 4 Occupational Medicine Unit, Fondazione Cà Granda, IRCCS Ospedale Maggiore Policlinico, Milan, Italy; 5 Dipartimento di Scienze del Farmaco, Università degli Studi di Padova, Padua, Italy; 6 IRCCS, Multimedica, Sesto San Giovanni (Milan), Italy; 7 Department of Neurosciences and Mental Health, Fondazione IRCCS Cà Granda Ospedale Maggiore Policlinico, Milan, Italy

**Keywords:** Beck Depression Inventory, Cardiovascular risk, Depression, Framingham risk score, Obesity, Proprotein Converatse Subtilisin/Kexin type 9

## Abstract

**Background:**

Depression and cardiovascular disease (CVD) are among the most common causes of disability in high-income countries, depression being associated with a 30% increased risk of future CV events. Depression is twice as common in people with diabetes and is associated with a 60% rise in the incidence of type 2 diabetes, an independent CVD risk factor. Proprotein convertase subtilisin/kexin type 9 (PCSK9), a key regulator of low-density lipoprotein cholesterol, has been related to a large number of CV risk factors, including insulin resistance. Aim of this study was to investigate whether the presence of depression could affect PCSK9 levels in a population of obese subjects susceptible to depressive symptoms and how these changes may mediate a pre-diabetic risk.

**Results:**

In 389 obese individuals, the Beck Depression Inventory (BDI-II) was significantly associated with PCSK9 levels. For every one-unit increment in BDI-II score, PCSK9 rose by 1.85 ng/mL. Depression was associated also with the HOMA-IR (homeostatic model assessment index of insulin resistance), 11% of this effect operating indirectly via PCSK9.

**Conclusions:**

This study indicates a possible mechanism linking depression and insulin resistance, a well-known CV risk factor, providing evidence for a significant role of PCSK9.

## Background

The extensive prevalence of cardiovascular diseases (CVDs) as a leading cause of mortality and morbidity worldwide [1] has repeatedly indicated that monitoring and correction of modifiable major CV risk factors (hypertension, smoking, dyslipidemia, diabetes and others) alone may not be sufficient to reduce the risk [2]. Lifestyle improvement has an important role in CV prevention and in particular excess body weight associates with a shorter lifespan and a significant increased risk of CV morbidity and mortality [3]. However, this condition may be a confounder for the less frequently evaluated clinical conditions linked to the CV risk, i.e. anxiety and depression [4]. Indeed, a reciprocal link exists between depression and obesity, a depressive mood increasing the odds for developing obesity by roughly 50% [5]. As estimated by the WHO, anxiety and depression are major CV risk factors and will become the second leading cause of disability in high income countries by 2030, carrying very high social and healthcare-related costs [6]. A number of meta-analyses have provided evidence of an association between clinical depression (or depressive symptoms) and CVD risk [7], the latter being raised by 30% in this condition [8].

Since the prognosis of patients with CVD and depression associates with a 2- to fourfold higher risk of subsequent events, an effect directly proportional to the depression severity [9], the screening and management of depression are strongly recommended for patients with CVD [10]. Up to 15–20% of patients with CVD suffer from depression and two-thirds of patients with myocardial infarction (MI) develop depression either concomitant with the event or during follow-up [11].

In this complex scenario, diabetes itself is an independent risk factor for CVD, leading to a rise of roughly two-fold on average, although the risk is subject to wide variations depending on the population [12]. Thus, it is worth mentioning that depression is twice as common in people with diabetes and associates with a 60% rise in the incidence of type 2 diabetes [13]. However, so far, although cross-sectional studies have shown a modest association between depression and high concentrations of haemoglobin A_1C_, one of the few prospective studies found a positive association between depression and the homeostasis model assessment-insulin resistance (HOMA-IR) values, an effect mostly mediated by central adiposity (reviewed in [14]).

Proprotein convertase subtilisin/kexin type 9 (PCSK9) has a pivotal role on the low-density lipoprotein cholesterol (LDL-C) levels by regulating the degradation of the LDL receptor (LDLR), although direct effects on additional atheroma components have been reported [15]. The ATHEROREMO-IVUS study showed that higher serum PCSK9 levels are linearly associated with a higher necrotic core fraction in coronary atheroma, regardless of LDL-C levels [16], confirming findings in PCSK9 KO mice, partially protected from neointimal formation [17]. In the context of CV prediction, PCSK9 concentrations, increased by insulin-resistance [18], are also associated with a raised susceptibility to MI, as assessed by genome association studies [19], although data from epidemiological studies are discordant [20–23]. The association between mood disorders and PCSK9 levels have received a significant contribution from a recent study on subjects affected by alcohol use disorder showing that PCSK9 cerebrospinal fluid levels are associated with the severity of behavioral disturbances [24].

The growing number of subjects affected by major depressive disorders worldwide and the possible association between depression and major adverse CV events [25] make it imperative to find markers predicting an enhanced CV risk. Depression and/or anxiety per se have been associated with a subclinical marker of atherosclerosis, i.e. the thickness of carotid intima-media [26], a sensitivity tool for the assessment of CV risk [27]. Among lipid biomarkers (total cholesterol, LDL-C and triglycerides) that have been associated with depression per se and depression severity, high levels of LDL-C seem to be those most related to depression [28, 29]. This evidence is in line with an increased risk of perceived depression in patients with familial hypercholesterolemia, characterized by elevated LDL-C levels and premature CV events [30].

Thus, considering that a depressive mood is a potential risk factor for type 2 diabetes and CVD, with no robust genetic support for a reverse causal effect of type 2 diabetes or CVD [31], primary aim of this study was to evaluate, in a population of obese subjects (more susceptible to depressive mood), the associations between depression, PCSK9 levels and HOMA-IR, a well-established CV risk factor [32]. In order to improve understanding of these relationships, the secondary objective was the application of the mediation analysis to ascertain if the effect of depression (independent variable) on HOMA-IR (dependent variable) was partially mediated by changes in PCSK9 levels (intervening variable).

## Subjects and methods

### Study design and participants

The baseline study population has been previously described [33]. We selected 389 obese subjects among participants of the cross-sectional SPHERE (Susceptibility to Particle Health Effects, miRNAs and Exosomes) study whose primary endpoint was to evaluate how air pollution exposure acted in overweight/obese individuals. Subjects were recruited from the Center for Obesity and Work-Activity (Fondazione IRCCS Ca’ Granda Ospedale Maggiore Policlinico in Milan, Lombardia, Italy). The eligibility criteria of the SPHERE study were: (1) older than 18 years at enrollment; (2) overweight/obese according to the following definitions: overweight, BMI between 25 and 30 kg/m^2^; obese: BMI of 30 kg/m^2^ or more; (3) resident in the Lombardy Region at the time of recruitment. Each participant provided written informed consent approved by the Ethics Committee of Fondazione IRCCS Cà Granda Ospedale Maggiore Policlinico (approval number 1425). On the day of recruitment, each subject underwent physical and anthropometric evaluations as well as cardiovascular and pulmonary function tests. The study was carried out over the period September 2010–April 2014.

### The Beck Depression Inventory

All participants were evaluated according to the Beck Depression Inventory II (BDI-II), considered as an appropriate tool to evaluate depressive symptoms in subjects with medical comorbidities such as obesity [34]. The following scores correspond to the different severity of depressive symptoms [34]: minimal range = 0–13, mild depression = 14–19, moderate depression = 20–28, and severe depression = 29–63.

### Clinical and laboratory measurements

Body weight and height were determined on a standard scale, waist circumference (WC) was measured at the umbilical level with subjects standing and breathing normally by the same physician at the end of the physical examination; BMI and weight to height ratio were also calculated. Systolic and diastolic blood pressures (SBP and DBP, respectively) were taken on the left arm using a mercury sphygmomanometer (mean of two measurements taken after 5 min of rest). Plasma lipids/lipoproteins and glucose were determined by established methodologies. C-reactive protein (CRP) and liver function tests (ALT, AST and GGT), as well as the full hematological profile (red blood cells, hematocrit and leukocyte formula) were determined. HbA1c was measured by ion-exchange high performance liquid chromatography on a VARIANT II Turbo Instrument (Glyco Hb Control, Menarini Diagnostics, Firenze, Italy); insulin by electrochemiluminescence immunoassay (ECLIA) on the Modular P automated analyser (Roche, Basel, Switzerland). HOMA-IR (homeostasis model assessment-insulin resistance) was calculated by means of fasting plasma glucose (mg/dL) times fasting plasma insulin (mU/L) divided by 405. Quantitative insulin sensitivity check index (QUICKI) is given by 1/[Log (fasting insulin, µU/mL) + Log (Fasting Glucose, mg/dL).

### PCSK9 evaluation (ELISA—Enzyme-Linked Immunosorbent Assay)

All patients underwent fasting blood sampling around 9 a.m, thus minimizing the possible confounding effect of circadian variations observed in PCSK9 levels. Plasma PCSK9 concentrations were measured by a commercial ELISA kit (R&D Systems, MN). Samples were diluted 1:20 and incubated onto a microplate pre-coated with a monoclonal human-PCSK9-specific antibody. Sample concentrations were obtained by a four-parameter logistic curve-fit, with a minimum detectable PCSK9 concentration of 0.219 ng/mL. Intra- and inter-assay CVs were 3.2% and 5.1%, respectively.

### Statistical analysis

Data were evaluated by standard descriptive statistics. Categorical variables were presented as absolute numbers and percentages. Continuous data were expressed as the mean ± SD or as the median and interquartile range (Q1-Q3), as appropriate. Normality and linearity assumptions were verified by graphical inspection. Univariate and multivariable linear regression models were used to test the relation between circulating PCSK9 levels and BDI-II score as the continuous predictor. Multiple analysis to test the association between PCSK9 concentrations and BDI-II was adjusted for a priori covariates (age, gender, BMI, smoking habit, antidepressant treatment) and for variables that were significantly related with PCSK9 in univariate analysis (*P* value < 0.05). Given the existence of multicollinearity among predictor variables (*i.e.* Total cholesterol and HDL cholesterol) the variance inflation factor (VIF) statistic was calculated. To determine the best performing model for evaluating the P-value, the VIF statistic as well as goodness of fit (R^2^) of several model equations, including one or more significant explanatory variables were used to predict PCSK9 levels. Finally, the best model selected to predict the association between circulating PCSK9 levels and BDI-II score was adjusted for: use of antidepressant treatment, age, gender, BMI, smoking habits, use of statin medications, non-HDL cholesterol, use of antihypertensive medications and triglycerides. We subsequently evaluated the association between depression score and the HOMA-IR, as adjusted for the above-mentioned selected covariates. The dependent variable, HOMA-IR, was log-transformed in order to achieve normality of models’ residuals. We performed causal mediation analysis to verify whether a third intermediate variable, *i.e.* PCSK9, is related to the observed exposure-outcome relationship. Indeed, a test of mediation examines whether the effect of the independent variable (x) on the dependent variable (y) occurs via a third, intervening variable (z). Linear regression coefficients were estimated by three equations:

- HOMA-IR =* intercept *+ **C∙**BDI-II + *e*, estimating the total effect of depression severity score on HOMA-IR.
- PCSK9 = *intercept *+ **a∙**BDI-II + *e*, estimating part of the indirect effect of depression severity score.
- HOMA-IR = *intercept *+ **b∙**PCSK9 + **c′∙**BDI-II + *e*, estimating part of the indirect effect and the direct effect of BDI-II on HOMA-IR.

Total effect **C** is the sum of direct effect (**c′**) and indirect effect (a*b). The indirect effect estimates the effect size of BDI-II score on HOMA-IR that is mediated by PCSK9. The use of antidepressant treatment, age, gender, BMI, smoking habit, use of statin medications, non-HDL cholesterol, use of antihypertensive medications and triglycerides were considered as confounders.

All statistical analyses were performed with SAS software (version 9.4; SAS Institute Inc., Cary, NC). The mediation analysis was carried out while utilizing the PROCESS program (model 4) provided by Hayes [35]. The Bootstrap confidence intervals (CIs) are provided with the number of bootstrap samples equal to 10,000.

## Results

### Study population

As shown in Table 1, the majority of the 389 subjects were females (68.9% vs 31.1%), with a mean BMI 33 ± 5.2 kg/m^2^ and large WC (mean ± SD: 100.7 ± 12.7 cm). Blood pressure was in the normal range, 29.1% of participants being on hypertensive medications. The case was similar for cholesterolemia, *i.e*. mean values of TC, LDL-C and non-HDL-C being in the upper range of normal (TC = 214.4 ± 41.1 mg/dL, LDL-C = 133.6 ± 36.2 mg/dL and non-HDL-C = 155.1 ± 41.0 mg/dL). Only 7.7% of participants were on statin medications. HDL-C and TG levels were also in the normal range: 59.0 ± 15.3 mg/dL and 117.2 ± 74.5 mg/dL, respectively. Levels of thyroid stimulating hormone (TSH) were normal (1.8 ± 1.1 U/mL) and no participant was on thyroid substitution/suppression therapies. As for other CV risk factors, 15.7% were current smokers and C-reactive protein (CRP) median level was 0.25 mg/L. There were no significant abnormalities in the standard laboratory tests including liver enzymes (AST and ALT), complete blood count (CBC) and white blood cell formula.

**Table 1:** Demographic and clinical characteristics of the studied population (n = 389)

Characteristics	Value
Age, years	50 ± 13
Gender	
Males	121 (31.1%)
Females	268 (68.9%)
WC, cm	100.7 ± 12.7
BMI, kg/m^2^	33.2 ± 5.4
Blood pressure	
Systolic	123.6 ± 15.5
Diastolic	77.2 ± 10.2
Total cholesterol, mg/dL	214.4 ± 41.1
HDL-C, mg/dL	59.0 ± 15.3
LDL-C, mg/dL	133.6 ± 36.2
Non-HDL-C, mg/dL	155.1 ± 41.0
Triglyceride, mg/dL	117.2 ± 74.5
C-reactive protein, mg/L	0.25 (0.12–0.52)
Glucose, mg/dL	93.1 ± 14.1
Glycated haemoglobin, mmol/mol	39.1 ± 5.7
Insulin level, U/mL	14.3 ± 8.4
AST, U/l	21.7 ± 9.0
ALT, U/l	26.6 ± 18.5
Gamma-glutamyltransferase, U/l	24.6 ± 17.8
TSH, U/mL	1.8 ± 1.1
Neutrophils, %	58 ± 7.8
Eosinophils, %	2.4 ± 1.5
Lymphocytes, %	31.5 ± 7.1
Monocytes, %	7.6 ± 2.4
Basophils, %	0.5 ± 0.3
Granulocytes, %	60.9 ± 7.3
Smoking status	
Never smoker	182 (46.8%)
Former smoker	141 (36.2%)
Current smoker	60 (15.5%)
Not avaliable	6 (1.5%)
Occupation	
Employee	240 (61.7%)
Unemployed	28 (7.2%)
Pensioner	83 (21.3%)
Housewife	26 (6.7%)
Not avaliable	12 (3.1%)

Continuous variables were expressed as mean ± standard deviation (SD) or as median [first quartile-third quartile], if not normally distributed

*ALT* alanine aminotransferase, *AST* aspartate aminotransferase, *BMI* body mass index, *HDL* high-density lipoprotein, *LDL* low-density lipoprotein, *TSH* thyroid-stimulating hormone, *WC* waist circumference

**Fig. 1:**
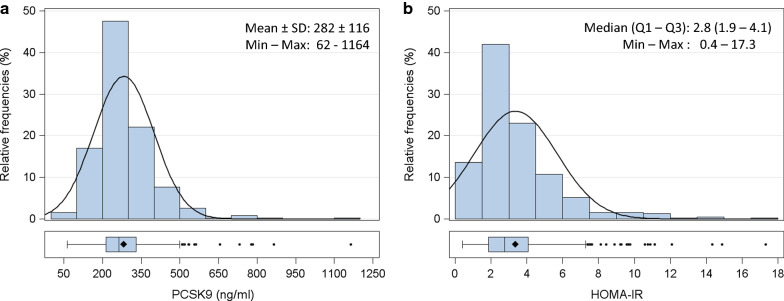
Distribution of fasting plasma concentrations of PCSK9 levels (ng/mL; panel **a**) and HOMA-IR (panel **b**) in 389 individuals. Data are presented as histograms and box-plots. PCSK9 is normally distributed, whereas HOMA-IR shows a skewed distribution. *PCSK9* pro-protein convertase subtilisin/kexin type 9, *HOMA-IR* homeostasis model assessment of insulin resistance. *Min* minimun, *Max* maximum, *SD* standard deviation

PCSK9 levels followed a Gaussian distribution (Fig. 1a) with mean levels of 282 ± 116 ng/mL. These values did not appear to be markedly different from those observed in prior studies by our group in the general population [36]. Glycemia and glycated hemoglobin were in the high normal range 93.1 ± 14.1 mg/dL and 39.1 ± 5.7 mmol/mol, respectively, as was the HOMA-IR. This last had a skewed distribution, with a median level of 2.8 (Q1–Q3: 1.9–4.1; Fig. 1b).

### Depression scores

As shown in Table 2, the mean Beck’s total score was 11.8 ± 8.6. BDI-II mean values were higher in women compared to men, *i.e.* 14 ± 8.7 and 6.9 ± 8.7, respectively (p < 0.0001). Less than 10% of the total sample received an antidepressant treatment, *i.e.* selective serotonin or norepinephrine/serotonin reuptake inhibitors (Additional file 1: Table S1). Antidepressant treatment was frequently prescribed to subjects with more severe depression, the therapeutic approach being quite homogeneously distributed among the different clusters of depression severity.

**Table 2:** Descriptive analysis of depressive symptoms (N = 389)

BDI-II scores	Value
Depression total score, Mean ± SD	11.8 ± 8.6
Depression total score by gender*, Mean ± SD	
Male	6.9 ± 6.0
Female	14 ± 8.7
Depression severity category	
Minimal (0-13)	240 (61.7%)
Mild (14–19)	78 (20.1%)
Moderate (20–28)	58 (14.9%)
Severe (≥ 29)	13 (3.3%)
Use of antidepressive drugs	
Yes	37 (9.5%)
No	352 (90.5%)

*BDI* Beck Depression Inventory

*P-value from t-test < 0.0001

### PCSK9 levels positively associate with BDI-II score and HOMA-IR

**Fig. 2:**
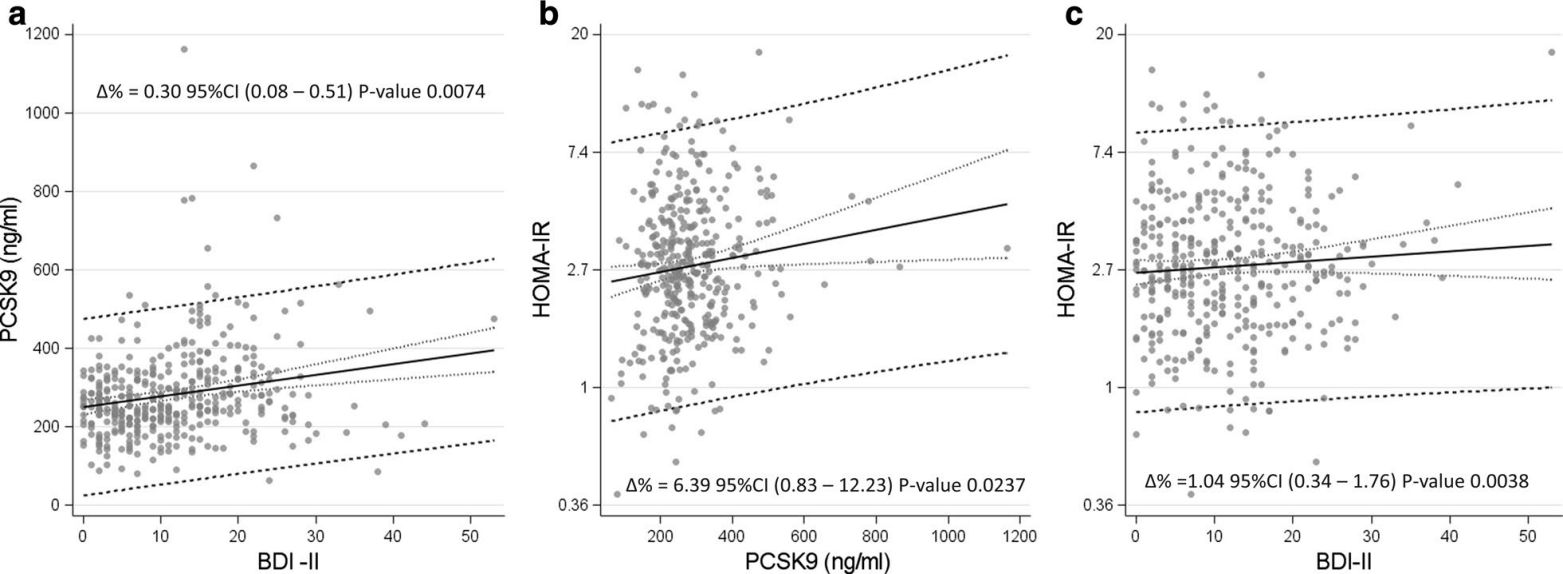
Scatterplots with regression line and confidence interval. Panel **a**—Association between severity of depression (BDI-II) and PCSK9. ∆ % represents the percentage increase in PCSK9 for one-unit increase in BDI-II score. Panel **b**–Association between PCSK9 and HOMA-IR on natural logarithmic scale, adjusted also for BDI-II. ∆ % represents the percentage increase in HOMA-IR for 100 ng/ml increase in PCSK9 concentration. Panel **c**—Association between severity of depression (BDI-II) and HOMA-IR on natural logarithmic scale. ∆ % represents the percentage increase in HOMA-IR for one-unit increase in BDI-II score. *BDI* Beck Depression Inventory, *HOMA*-*IR* homeostasis model assessment of insulin resistance, *PCSK9* pro-protein convertase subtilisin/kexin type 9. Beta regression coefficients of panel **a** and **b** were used to estimate indirect effect of BDI-II on HOMA-IR

In the univariate analysis the severity of depression (BDI-II score) was positively associated with PCSK9 circulating levels with a rise of 0.44% (β = 2.75, Table 3) for every one-unit increase of BDI-II (Δ % = 0.44, 95%CI 0.23–0.65, p < 0.0001). The strength of this association was confirmed in multivariable analysis, *i.e.* for every one-unit increment in BDI-II score, PCSK9 rose by 0.3% (β = 1.85, Δ % = 0.30, 95%CI 0.08–0.51, p = 0.0074; Fig. 2a), after correction for variables that in univariate analysis were associated with PCSK9 and were not collinear among them (Additional file 1: Table S2). Relative to the association between PCSK9 and HOMA-IR, in this cohort, a statistical 6.39% rise of the HOMA-IR for every 100 ng/mL increment in PCSK9 levels (β = 0.00619, Δ % = 6.39 95%CI: 0.83–12.23, P-value 0.0237) was observed (Fig. 2b).

**Table 3:** Linear regression models to evaluate the association of PCSK9 levels with severity of depression (BDI-II score)

	β	SE	P-value
Univariate model			
Intercept	249.98	9.85	<.0001
BDI-II Score (for unit increase)	2.75	0.68	<.0001
Adjusted model			
Intercept	188.43	53.14	0.0004
BDI-II Score (for unit increase)	1.85	0.69	0.0074
Age, years	− 0.07	0.48	0.8824
Gender			0.0004
Male	− 46.10	12.86	
Female	Ref		
BMI	−0.29	1.00	0.7673
Smoking status			0.0157
Current smoker	21.29	15.67	0.1752
Former smoker	34.13	11.88	0.0043
Never smoker	Ref		
Non-HDL, mg/dl	0.49	0.15	0.0015
Statin medications			
Yes	63.35	21.22	0.0022
No	Ref		
Triglyceride, mg/dl	0.07	0.08	0.3730
Antihypertensive medications			
Yes	13.07	13.9	0.3479
No	Ref		
Antidepressive medications			
Yes	0.34	18.02	0.9848
No	Ref		

*BDI* Beck Depression Inventory, *BMI* body mass index, *HDL* high-density lipoprotein, *PCSK9* pro-protein convertase subtilisin/kexin type 9, *Ref* stands for reference value

### Causal mediation

**Fig. 3:**
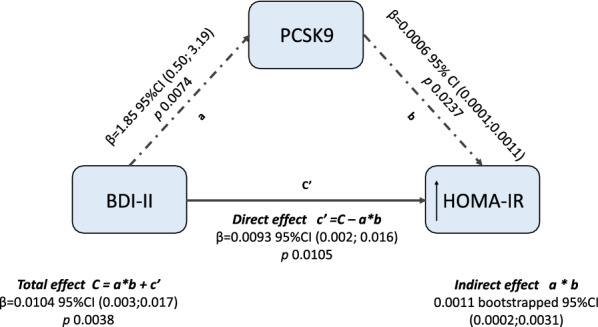
Conceptual diagram of causal mediation analysis, that hypothesized mechanism linking BDI-II and HOMA-IR. The solid black arrow represents the effect of BDI-II on HOMA-IR levels that operates directly or through a pathway different from the mediator analyzed in the current study (PCSK9). The dotted black arrows represent the suggested alternative pathway, where an indirect effect of BDI-II on HOMA-IR is mediated by PCSK9 levels. The black thin arrows indicate increased levels of HOMA-IR. *BDI* Beck Depression Inventory, *HOMA-IR* homeostasis model assessment of insulin resistance, *PCSK9* pro-protein convertase subtilisin kexin type 9

The results of mediation analysis suggested that the association between HOMA-IR and BDI-II score is partially mediated by PCSK9 levels. The analysis showed that the BDI-II score has a significant overall effect on HOMA-IR (total effect C, Δ % = 1.04, 95%CI 0.34–1.75, p = 0.0038; Figs. 2c and 3). The direct effect of the BDI-II on HOMA-IR was significant (direct effect c′, Δ % = 0.93, 95% CI 0.22–1.65, p = 0.0105, Fig. 3). Another part of such effect may operate through PCSK9 (indirect effect C- c′, Δ % = 0.11 bootstrapped 95%CI 0.02–0.31, Fig. 3), Consequently, 11% of the total effect of BDI-II on HOMA-IR takes place via PCSK9.

## Discussion

In the still ongoing debate on the bidirectional association between major depressive disorders and type 2 diabetes, as well as between depression and CVD risk [31], the present report on 389 obese individuals, mainly females, supports the following conclusions: (1) depressive symptoms, as assessed by BDI-II score, seem to raise PCSK9 levels, (2) depressive symptoms directly associate with HOMA-IR, a pre-diabetes related CV risk variable [32], and (3) 11% of such an effect operates via an indirect path, through PCSK9. Reliability of these conclusions is based on the following the three pillar criteria: (a) statistically significant association between depression and outcome (HOMA-IR); (b) exposure (BDI-II score) must have an effect on the mediator (PCSK9), (c) the mediator has to be associated with the outcome (HOMA-IR) when exposure is controlled, *i.e.*, after adjusting for PCSK9 levels.

### Obesity and depression

Since earlier studies indicated that depression and obesity often come hand in hand, the relationship between the two is difficult to tease apart. Data from a Mendelian randomization analysis provided evidence that a higher BMI is likely to have a causal role in determining the likelihood of an individual developing depression [37]. In our cohort, a trend toward a positive association between BMI and depressive symptoms (β = 0.121, p = 0.13) was found, after correcting for age and gender. The complex biological profile of depression does not appear to be clearly linked to definite genetic variants. A more informative approach may take into account life habits and psychological risk factors, eventually leading to altered hypothalamic responses to metabolic signaling molecules [38]. Findings from the present report, advocating the potential clinical significance of the association between depression and PCSK9 in mediating the rise in HOMA-IR, indicate that plain evaluation of the anthropometric variables may not be adequate for a full explanation of the PCSK9 changes.

### Depression, CVD risk and HOMA-IR

In order to identify individuals depressed or at high risk of depression with a potential CV risk, a number of screening tests are now available [39]. These can be applied successfully also to individuals who may be just at CV risk. Aside from the widely reported questionnaires, commonly used for the screening of depression, such as the PHQ-2 [40] of some value in order to evaluate large numbers of individuals, the more classical and sensitive tests are of better help in order to obtain a baseline assessment of symptom severity and to monitor subsequent improvement on antidepressant treatment. In the present report, the classical BDI was selected, as widely applied also in earlier studies in cardiac patients [41, 42]. More specifically the BDI-II, as per the 1996 improved version [43] can be successfully administered both to adolescents and adults, as a tool of medical assistance but also for research. The BDI-II lists 21 symptoms, with item responses scoring from 0 to 3 and total scores ranging from 0 to 63. Considering that depression is approximately twice as prevalent in women [44], in our cohort BDI-II mean values were significantly higher in women compared to men, *i.e.* 14 and 6.9, respectively [45].

Among the reported mechanistic links between depression and CVD [7], conditions characterized by insulin resistance have been pointed out as sharing pathogenic mechanisms [46]. This evidence is in line with our findings showing a direct and significant effect of the BDI-II on HOMA-IR. Evaluation of the HOMA-IR provides a sensitive biomarker of CVD risk, particularly in the forecasting of coronary atherosclerosis, independent of established risk factors, including hsCRP [32], as also confirmed in a large series of Italian type 2 diabetics [47].

### Depression and PCSK9

Although it is well established that PCSK9 was discovered in the brain as the human equivalent of NARC-1 (Neural Apoptosis Regulated Convertase-1) implicated in the differentiation of cortical neurons [48], its relationship with the depressive mood has not been clarified as yet [49, 50]. Albeit we found a positive association between BDI-II score and PCSK9, a mutual effect cannot be excluded. While genetic analyses found a positive association between raised risk of depressive mood and circulating levels of PCSK9, the notion that lipid-lowering drugs might increase the risk of depression was not confirmed in trials with PCSK9 inhibitors [51]. Among 479,522 individuals of UK Biobank, those carrying the LDL-lowering T allele variant rs1159147 of the *PCSK9* gene, had a concomitant higher risk of type 2 diabetes and depression [52]. A 19% increased risk of depression was also found among carriers of SNPs related to PCSK9 inhibition [53]. Conversely, data from FOURIER (Further Cardiovascular Outcomes Research with PCSK9 Inhibition in Subjects with Elevated Risk) with evolocumab reported that rates of depressive disorders were 2.0% in each study group, events of major depression being more frequent in patients on placebo (14 events) than in those given evolocumab (5 events) [54, 55]. Similar conclusions were reached among individuals with type 2 diabetes and CVD given alirocumab who did not report any depressive symptom, despite a large reduction in atherogenic lipoproteins and LDL particle numbers [56].

### PCSK9 and CVD risk

Concerning the relationship between PCSK9 and CV risk, although some studies on PCSK9 levels have not reported any association with CV events [20, 21], other populations, meta-analyses and genome-wide association studies have instead supported the predictivity of PCSK9 levels in CV events [23, 57, 58]. Thus, considering that depression is associated with a 30% increased risk of future CV events [8], the reported positive association between BDI-II score and the rise in PCSK9 levels becomes of interest. A genetic reduction of PCSK9 levels by 50% is associated with a similar percent reduction of coronary heart disease (CHD) risk [59] and a genetic large-scale study on 337,536 individuals of British ancestry reported that the rs11591147 *PCSK9* mutation had a protective effect not only on hyperlipidemia, but also on the risk of CHD (− 27%) and ischemic stroke (− 39%) [60]. A further hypothesis linking PCSK9 to atherosclerosis is the direct role that this protein can play on atherogenesis (reviewed in [61]). Indeed, PCSK9, expressed in vascular smooth muscle cells, human atherosclerotic plaques and epicardial adipose tissue [62], reduces macrophage cholesterol efflux capacity, raises the migratory ability of monocytes and the expression of scavenger receptors, thus enhancing ox-LDL uptake in monocytes and macrophages.

### PCSK9 and insulin resistance

Besides raised PCSK9 levels, an enhanced CV risk, in our cohort, may be consequent to the increased incidence of insulin resistance and diabetes mellitus. Several studies have shown that insulin, HOMA-IR and HbA1c are correlated with PCSK9 (reviewed in [49]). The link between PCSK9 and insulin resistance has been confirmed in obese individuals undergoing gastric bypass. Roux-en-Y gastric bypass promotes a significant reduction in plasma PCSK9 levels, changes apparently more significantly associated with improvement of glucose rather than lipid homeostasis [63]. Very recently, in severely obese patients undergoing bariatric surgery, our group confirmed a pivotal role played by adipose tissue and insulin resistance on PCSK9 levels [64]. This evidence was confirmed in the Dallas Heart Study, reporting that PCSK9 levels were significantly associated with glucose metabolism, including HOMA-IR [65]. These findings have been partially confirmed in prospective cohort studies in which plasma PCSK9 levels were positively correlated with HOMA-IR, but not with glucose homeostasis [66]. Finally, in patients with stable CVD, low PCSK9 plasma levels were associated with a metabolic pattern characterized by low HDL cholesterol, the metabolic syndrome, elevated BMI, insulin resistance and diabetes with diffuse non-obstructive coronary atherosclerosis [67].

### Limitations

The present study, conducted in a large number of subjects, has definite limitations, *i.e.* being a retrospective analysis in obese patients with no diagnosis of prediabetes or diabetes but with BDI-II scores available at recruitment. Thus, in order to limit possible comorbidities linked to obesity, a priori we selected the healthiest participants and further corrected for possible confounders. Moreover, only a small proportion of those classified with depression were taking psychotropic medications, similar to prior observations in the EUROASPIRE cardiac patients [68]. This may partly be due to our assessment of current, but not previous, symptoms and of the use of psychotropic medications; in addition, a large proportion of those with depression were likely to have subclinical or mild symptomatology.

## Conclusions

Among the effects of common modifiable risk factors on CVD and mortality, symptoms of depression are commonly listed. In view of the common occurrence of diabetes linked life-habits with depression [69], the evaluation of PCSK9 may thus offer a pathophysiological link, being associated with an insulin resistance marker, predictor of a raised CV risk. Since depression is highly prevalent in patients with CVD and portends adverse CV outcomes and increased healthcare costs, the identification of a biomarker linked to depression that correlates with insulin resistance may identify people who could benefit most by targeted interventions [70].

## Supplementary information

**Additional file 1: Table S1.**Antidepressant treatments (n=37).** Figure S1.** Slope coefficients from univariate linear regression models evaluating the association between levels of PCSK9 and characteristics of subjects.

## Data Availability

The data will be available on request due to privacy/ethical restriction. The data that support the findings of this study are available on request from the corresponding author.

## Abbreviations

BDI-II: Beck Depression Inventory
BMI: Body Mass Index
CI: Confidence interval
CRP: C-reactive protein
CVD: Cardiovascular disease
HDL: High-density lipoprotein
HOMA-IR: Homeostasis model assessment of insulin resistance
ELISA: Enzyme-linked immunosorbent assay
FRS: Framingham Risk Score
LDL: low-density lipoprotein
NARC-1: Neural Apoptosis Regulated Convertase-1
PCSK9: Proprotein convertase subtilisin/kexin type 9
QUICKI: Quantitative insulin sensitivity check index
SD: Standard deviation
TC: Total cholesterol
TG: Triglycerides
WC: Waist circumference

## Supplementary information

**Supplementary information** accompanies this paper at 10.1186/s12933-020-01158-6 .
