# Supplementary material for: Depression and cardiovascular risk—association among Beck Depression Inventory, PCSK9 levels and insulin resistance
Source: Cardiovasc Diabetol. 2020 Nov 3;19:187. doi: 10.1186/s12933-020-01158-6 (PMC7641831; doi:10.1186/s12933-020-01158-6)
Supplement: Supplementary file 1 — Additional file 1: Table S1.Antidepressant treatments (n=37). Figure S1. Slope coefficients from univariate linear regression models evaluating the association between levels of PCSK9 and characteristics of subjects. [file 12933_2020_1158_MOESM1_ESM.docx]

**Supplemental Tables**

**Supplemental table 1**. Antidepressant treatments (n=37)

| **Drug** | **N (%)** |
| --- | --- |
| Amitriptyline | 1 (2.7%) |
| Bupropion | 1 (2.7%) |
| Citalopram | 4 (10.8%) |
| Duloxetine | 2 (5.4%) |
| Escitalopram | 1 (2.7%) |
| Fluoxetine | 7 (18.9%) |
| Fluvoxamine | 2 (5.4%) |
| Paroxetine | 5 (13.6%) |
| Sertraline | 7 (18.9%) |
| Venlafaxine | 7 (18.9%) |

**Supplemental table 2.** Slope coefficients from univariate linear regression models evaluating the association between levels of PCSK9 and characteristics of subjects.

| **Independent variable** |  | **β** | **SE** | **P-value** | | **R^2^** |
| --- | --- | --- | --- | --- | --- | --- |
| **Age, years** |  | **1.28** | **0.45** | **0.0047** | | **0.0205** |
| **Gender** |  |  |  |  |  |  |
| ***Females*** |  | **45.91** | **12.55** | **0.0003** | | **0.0334** |
| ***Males*** |  | **REF** | **-** |  |  |  |
| Waist circumference, cm |  | -0.04 | 0.47 | 0.9397 | | 0.0004 |
| BMI, *kg/m^2^* |  | -0.01 | 1.1 | 0.9905 | | 0.0001 |
| **Smoking status** |  |  |  |  |  |  |
| ***Never smoker*** |  | **REF** | **-** | **-** | **0.0501** | **0.0156** |
| ***Former smoker*** |  | **31.69** | **13.04** | **0.0156** |  |  |
| ***Current smoker*** |  | **19.08** | **17.3** | **0.2709** |  |  |
| Occupation |  |  |  |  |  |  |
| *Employee* |  | REF | - | - |  | 0.0066 |
| *Unemployed* |  | 20.64 | 23.41 | 0.3786 | 0.4807 |  |
| *Pensioner* |  | 21.14 | 14.93 | 0.1576 |  |  |
| *Housewife* |  | 11.19 | 24.20 | 0.6441 |  |  |
| Blood pressure, *mmHg* |  |  |  |  |  |  |
| *Sistolic* |  | -0.14 | 0.38 | 0.7043 | | 0.0004 |
| *Diastolic* |  | 0.16 | 0.58 | 0.7825 | | 0.0002 |
| **Antihypertensive medications** |  |  |  |  |  |  |
| ***Yes*** |  | **31.64** | **12.92** | **0.0148** | | **0.0153** |
| ***No*** |  | **REF** | **-** |  |  |  |
| C-reactive protein, *mg/l* |  | 3.68 | 6.63 | 0.5798 | | 0.0008 |
| **Total cholesterol, *mg/dl*** |  | **0.61** | **0.13** | **<0.0001** | | **0.0545** |
| HDL-C, *mg/dl* |  | 0.61 | 0.36 | 0.0934 | | 0.0074 |
| **LDL-C, *mg/dl*** |  | **0.58** | **0.15** | **0.0001** | | **0.0377** |
| **non-HDL-C, *mg/dl*** |  | **0.51** | **0.14** | **0.0004** | | **0.0325** |
| **Statin medications** |  |  |  |  |  |  |
| ***Yes*** |  | **58.56** | **21.96** | **0.008** | | **0.018** |
| ***No*** |  | **REF** | **-** |  |  |  |
| **Triglyceride, mg/dl** |  | **0.20** | **0.07** | **0.0066** | | **0.0193** |
| AST, *U/I* |  | -0.20 | 0.61 | 0.7436 | | 0.0003 |
| ALT, *U/I* |  | -0.51 | 0.30 | 0.0876 | | 0.0077 |
| Gamma-Glutamyltransferase, *IU/l* |  | 0.09 | 0.31 | 0.7651 | | 0.0002 |
| TSH, *mIU/l* |  | 5.48 | 5.14 | 0.2874 | | 0.0030 |
| Glucose, *mg/dl* |  | 0.60 | 0.39 | 0.1256 | | 0.0062 |
| Glycated hemoglobin, *mmol/mol* |  | 0.87 | 0.97 | 0.3728 | | 0.0021 |
| Insulin level, *U/ml* |  | 0.87 | 0.66 | 0.1872 | | 0.0046 |
| Neutrophil,*. %* |  | -0.53 | 0.72 | 0.4649 | | 0.0014 |
| Eosinophils, *%* |  | 4.94 | 3.78 | 0.1919 | | 0.0045 |
| Lymphocytes, *%* |  | 0.64 | 0.79 | 0.4205 | | 0.0017 |
| Monocytes, *%* |  | -1.87 | 2.35 | 0.4281 | | 0.0017 |
| Basophils, *%* |  | -7.94 | 19.81 | 0.6886 | | 0.0004 |
| Granulocytes, *%* |  | -0.40 | 0.76 | 0.6005 | | 0.0007 |
| Antidepressive medications |  |  |  |  |  |  |
| *Yes* |  | 24.19 | 20.11 | 0.2299 | | 0.0037 |
| *No* |  | REF | - |  |  |  |

ALT: alanine aminotransferase; AST: aspartate aminotransferase; BMI: body mass index; HDL: high-density lipoprotein; LDL: low-density lipoprotein; PCSK9: proprotein convertase subtilisin/kexin type 9; TSH: thyroid-stimulating hormone; WC: waist circumference. REF stands for reference value.
